# Quantitative Determination of Nitrofurazone Metabolites in Animal-Derived Foods Based on a Background Fluorescence Quenching Immunochromatographic Assay

**DOI:** 10.3390/foods10071668

**Published:** 2021-07-20

**Authors:** Yuping Wu, Jia Wang, Yong Zhou, Yonghua Qi, Licai Ma, Xuannian Wang, Xiaoqi Tao

**Affiliations:** 1 College of Life Science and Basic Medicine, Xinxiang University, Xinxiang 453003, China; wuyuping62@xxu.edu.cn (Y.W.); qyh@xxu.edu.cn (Y.Q.); 2 College of Food Science, Southwest University, Chongqing 400715, China; wangjia2020@cau.edu.cn; 3 College of Veterinary Medicine, China Agricultural University, Beijing 100193, China; zhouyong@cau.edu.cn; 4 Beijing WDWK Biotech Co., Ltd., Beijing 100095, China; malicai@wdwkbio.com

**Keywords:** semicarbazide (SEM), background fluorescence-quenching immunochromatographic assay (bFQICA), quantitative determination, animal-derived foods

## Abstract

Due to their facile synthesis and friendly functionalization, gold nanoparticles (AuNPs) have been applied in all kinds of biosensors. More importantly, these biosensors, with the combination of AuNPs and immunoassay, are expected to be used for the detection of different compounds with low concentrations in complex samples. In this study, a AuNPs-labeled antibody immunoprobe was prepared and combined with a fluorescence-quenching principle and a background fluorescence-quenching immunochromatographic assay (bFQICA), achieving rapid on-site detection. By using a portable fluorescence immunoquantitative analyzer and a QR code with a built-in standard curve, the rapid quantitative determination for nitrofurazone metabolite of semicarbazide (SEM) in animal-derived foods was realized. The limits of detection (LODs) for bFQICA in egg, chicken, fish, and shrimp were 0.09, 0.10, 0.12, and 0.15 μg kg^−1^ for SEM, respectively, with the linear range of 0.08–0.41 μg L^−1^, the recoveries ranging from 73.5% to 109.2%, and the coefficient of variation <15%, only taking 13 min for the SEM detection. The analysis of animal-derived foods by bFQICA complied with that of liquid chromatography-tandem mass spectrometry (LC-MS/MS).

## 1. Introduction

As a broad-spectrum antibiotic, nitrofurazone (NFZ) is a well-known member of the nitrofurans class and is widely applied in husbandry to prevent and control a variety of animal diseases caused by *Salmonella* and *Escherichia coli* infection [1,2]. Meanwhile, NFZ was also used as a medicinal feed additive to prevent the dysentery and bacterial enteritis in swine. NFZ as a kind of commonly used drug, can be metabolized to SEM in an animal’s body [3]; therefore, the detection of SEM is usually used to reflect the residual state of NFZ. Studies have found that, after a period of accumulation in the human body, nitrofuran metabolites can lead to various organ diseases and can cause serious harm to human health, such as irreversible damage to the central nervous system, liver, kidney, heart, hypothalamus, reproductive system, and so on; toxic and side effects; allergic reaction or allergy; bacterial drug resistance; and dysbacteriosis, teratogenesis, carcinogenesis, and mutagenesis [4,5]. Since 1995, the European Union has prohibited nitrofuran use in livestock, aquaculture, and poultry [6]. Moreover, China and USA have also strictly prohibited nitrofuran application in food-producing animals [7,8]. The European Union and the USA have set the minimum required performance limit (MRPL) (1.0 μg kg^−1^) for SEM in animal-derived foods [9]. Hence, it is essential to establish effective methods for the detection of SEM in animal-derived foods.

Indeed, various methods have been established for detecting NFZ and/or SEM (the metabolite of NFA) in animal-derived foods, such as high-performance liquid chromatography-ultraviolet (HPLC-UV) [10], HPLC with fluorescence (HPLC-FLD) [11], and HPLC-tandem mass spectrometry (HPLC-MS/MS) [12,13,14,15,16]. However, the above instrumental methods require professional knowledge of operators and costly instruments, and they are unsuitable for on-site detection, which limits their use. The immunoassay is a rapid useful technique for SEM analysis with high throughput tests, such as enzyme-linked immunosorbent assay (ELISA) [17] and fluorescence-linked immunosorbent assay (FLISA) [18]. However, ELISA and FLISA are heterogeneous reactions and time-consuming, which requires tedious washing steps. The colloidal gold immunochromatographic assay (CGICA) [19] is simple, fast, and low cost; however, it always shows the disadvantages of just a qualified determination with a relatively high detection limit.

Due to their facile synthesis and friendly functionalization, gold nanoparticles (AuNPs) have been applied in all kinds of biosensors [20], whether chemical and biological, drug delivery, or photothermal therapy [21,22,23]. More importantly, these biosensors with the combination of AuNPs and immunoassay are expected to be used for the detection of different compounds with low concentrations in complex samples [24,25,26]. Wu et al. developed a background fluorescence-quenching immunochromatographic assay (bFQICA) for the detection chloramphenicol (CAP) and aflatoxin M_1_ (AFM_1_) in milk with the limit of detection (LOD) for CAP of 0.0008 μg L^−1^ and for AFM_1_ of 0.0009 μg L^−1^ [25]. In 2020, we successfully conducted the bFQICA to achieve co-determination of quinoxaline-2-carboxylic acid (QCA) and 3-methyl-quinoxaline-2-carboxylic acid (MQCA) in pork, with a sensitivity of 0.1–1.6 μg L^−1^ and only taking 30 min for the detection, exhibiting convenience and efficiency [26]. The bFQICA has the advantages of having high specificity and high sensitivity, and it is quantitative, portable, and accommodates direct read-out mini devices; but so far, there has been no report on SEM detection by the bFQICA.

**Figure 1:**
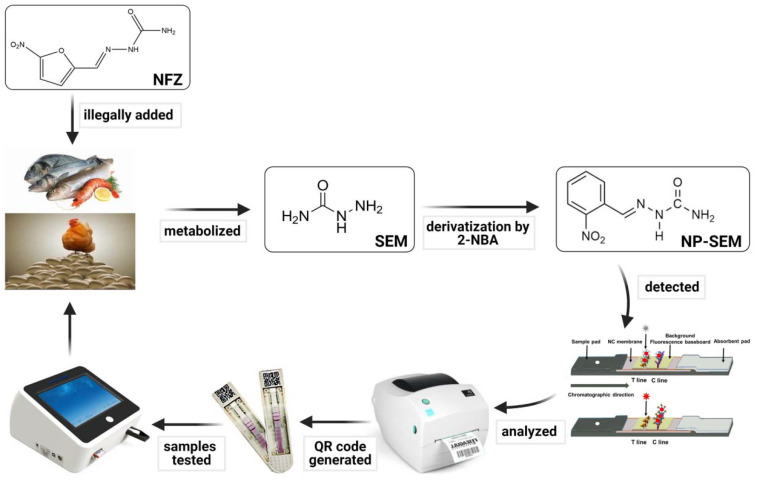
The scheme of bFQICA for detection of SEM in animal-derived foods.

In this study, a bFQICA, achieving on-site quantitative determination of SEM residues in animal-derived foods (egg, chicken, fish, and shrimp) was established (Figure 1), in which AuNPs were used to quench the fluorescence of a background fluorescence baseboard, and a portable fluorescence immunoquantitative analyzer was used to measure the background fluorescence.

## 2. Materials and Methods

### 2.1. Chemicals and Equipment

The parent nitrofurans and SEM were obtained from Dr. Ehrenstorfer (Augsburg, Germany), and other related materials can be seen in the Supplementary Materials. CPSEM-OVA (carboxybenzaldehyde semicarbazone-ovalbumin, 5.47 mg mL^−1^) and anti-NPSEM monoclonal antibody (mAb) (4.05 mg mL^−1^) were obtained from Beijing WDWK Biotech Co., Ltd. (Beijing, China). Goat anti-mouse IgG was obtained from Jackson ImmunoResearch Laboratories, Inc. (West Grove, PA, USA).

The sample pad and absorbent pad were from Shanghai Liangxin Co., Ltd. (Shanghai, China). The background fluorescence baseboard was obtained from Shanghai Xinpu Biotechnology Co. Ltd. (Shanghai, China). A fluorescence immune-quantitative analyzer was from Simp Bio-Science Co., Ltd. (Shanghai, China), and the UV-Vis spectrophotometer was obtained from Hitachi Ltd. (Tokyo, Japan). The soft of NiceLabel Pro 2017 was obtained from NiceLabel China (Shanghai, China)

### 2.2. Preparation and Characterization of AuNPs-Labeled Antibody Immunoprobe

The preparation of the AuNPs-labeled antibody (AuNPs-anti-NPSEM mAb) immunoprobe was according to previous literature with slight modifications [26,27,28].

First, AuNPs were synthetized by the reduction method of trisodium citrate [29].

Second, for the preparation of AuNPs-anti-NPSEM mAb, the pH of AuNPs (1 mL) was adjusted to 8.0 (K_2_CO_3_, 0.1 M), then the amount of anti-NPSEM mAb was added, quickly mixed, and incubated for 10 min at room temperature (RT). Afterward, 20 µL of BSA (20%, w/v) was added, mixed for blocking, and the mixture was centrifuged (8000 rpm, 10 min, 4 °C). Finally, the supernatant was quickly moved, and the pellet was diluted in storage buffer (200 µL). In addition, AuNPs-anti-NPSEM mAb (4 µL) was transferred into a microplate well and ultrasonically resuspended, then stored for use (4 °C).

### 2.3. Preparation of bFQICA Strip

The bFQICA strip contained a sample pad, background fluorescence baseboard, NC membrane, absorbent pad, and background fluorescence baseboard. Initially, CPSEM-OVA was dissolved in 0.02 M PBS with the final concentrations of 0.17 mg/mL and sprayed onto the NC membrane to form test line (T line). Goat anti-mouse IgG was dissolved in PB (0.02 M) with the final concentrations of 0.33 mg/mL and sprayed on the NC membrane as the control line (C line). The spraying amount of CPSEM-OVA and goat anti-mouse IgG was 0.7 µL/cm, with an interval between the T line and C line of 3.00 mm. Then, the as-prepared NC membrane was dried at 45 ℃ for 2 h. Next, the NC membrane was attached to the fluorescent region of the background fluorescence baseboard; the sample pad and absorbent pad were assembled on the two sides of the background fluorescence baseboard, respectively. Then, on the NC membrane and the assembled background fluorescence baseboard with a 2 mm overlap, the strip was cut into 4.72 mm wide test strips. Finally, all was put into a jam case and assembled into a bFQICA strip, and the assembled strips were stored and kept sealed in a dry environment until use.

### 2.4. The Procedure of bFQICA for SEM

First, a standard or samples extraction solution (200 µL) was added to the freeze-dried AuNPs-anti-NPSEM mAb immunoprobes, was gently blown by the pipette, and was mixed until the purplish red particles at the bottom of the well were completely dissolved, after which the solution was incubated for 3 min at RT in microplate well. After that, the above mixture (120 µL) was added into the sample pad. As a result, the mixture could move toward the absorbent pad through capillarity. Finally, the strip was measured by the fluorescence immune-quantitative analyzer after 10 min incubation at RT, and the fluorescence signals for (F_1_/F_2_) T/C lines were measured.

### 2.5. Standard Curves and Generation of QR-Code

For the quantitative assay, four parameters were input into software (Nice Label Pro 2017) to generate QR-code with the built-in standard curve, and the QR-code was printed by barcode printer (Label Shop). The accurate concentration of analytes could be obtained by scanning the QR-code (Supplementary Materials).

### 2.6. Sample Pretreatment

The animal-derived foods (egg, chicken, fish, and shrimp) were from Xinxiang local supermarkets and were stored at −20 °C before use. The sample pretreatment was similar to our previous method [26] (Supplementary Materials). Before the detection by bFQICA, the collected solution had a dilution factor of 5, with a sample diluent (0.02 M PBS containing 0.05% Tween-20, pH 7.4) to remove the matrix interference.

### 2.7. Validation of bFQICA

Because of the low molecular weight of SEM, 2-NBA is often used to derivatize the metabolite to increase the molecular weight in the sample pretreatment process before detection. For validation of bFQICA, animal-derived food samples were confirmed to be SEM-free by LC-MS/MS (Supplementary Materials).

## 3. Results and Discussion

### 3.1. Principle of bFQICA for Quantification of SEM

**Figure 2:**
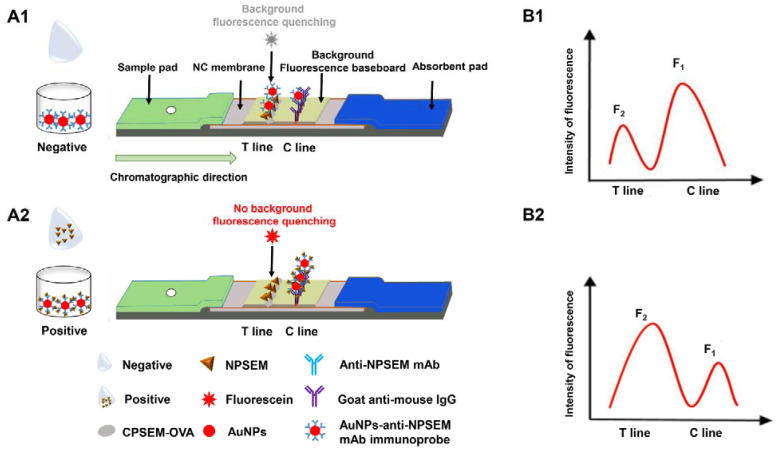
Scheme of bFQICA for the quantitative detection of (NP)SEM. (**A**): The diagram of bFQICA test card, when there was no (NP)SEM (negative) (**A1**), or when there was in the presence of (NP)SEM (positive) (**A2**); (**B**): the fluorescence of C line (F_1_) and T line (F_2_), when there was no (NP)SEM (negative) (**B1**), or when there was in the presence of (NP)SEM (positive) (**B2**).

The detection mode of this study was competitive reaction. The background fluorescence of the membrane strip and the relative fluorescence intensity of the T line were detected quantitatively. AuNPs-anti-NPSEM mAb immunoprobes were bound with NPSEM in the standard or samples extraction solution, and then the mixture was dripped onto the sample pad, moving toward the absorbent pad through capillarity. As shown in Figure 2(A1), when there was no (NP)SEM (negative), the immunoprobes (AuNPs-anti-NPSEM mAb) bound with the CPSEM-OVA coated on the T line in the NC membrane, which could obviously quench (cover) the fluorescence of the T line (F_2_) generated from the fluorescein of the background fluorescence baseboard. The remaining immunoprobes (AuNPs-anti-NPSEM mAb) continued to move toward the C line and were bound with the goat anti-mouse IgG, generating less fluorescence at the C line (F_1_) due to the quenching (covering) of the fluorescein of the background fluorescence baseboard by AuNPs, in which the ratio of F_1_/F_2_ was maximum (max) (Figure 2(B1)).

Conversely, when (NP)SEM (positive) was present (Figure 2(A2)), the immunoprobes (AuNPs-anti-NPSEM mAb) were bound with the analytes, and then fewer immunoprobes (AuNPs-anti-NPSEM mAb) would bind with the CPSEM-OVA coated on the NC membrane, with less of a quenching (covering) effect, thus generating more fluorescence on the T line (F_2_). Moreover, these probes (the unbound immunoprobes (AuNPs-anti-NPSEM mAb) and AuNPs-anti-NPSEM mAb-analytes complex) could be captured by the goat anti-mouse IgG on the C line and an additional quenching (covering) effect occurred, with the less fluorescence of the C line (F_1_), in which the ratio of F_1_/F_2_ was minimum (min) (Figure 2(B2)). As the concentration of (NP)SEM increased, the ratio of F_1_/F_2_ decreased. Furthermore, F_1_ waned and F_2_ waxed with the increased concentration of (NP)SEM. In addition, the concentration of (NP)SEM could be directly displayed by the built-in QR-code, which only took 13 min for the detection of (NP)SEM, including 10 min of incubation and 3 min of signal collection and data calculation.

### 3.2. Characterization of AuNPs

**Figure 3:**
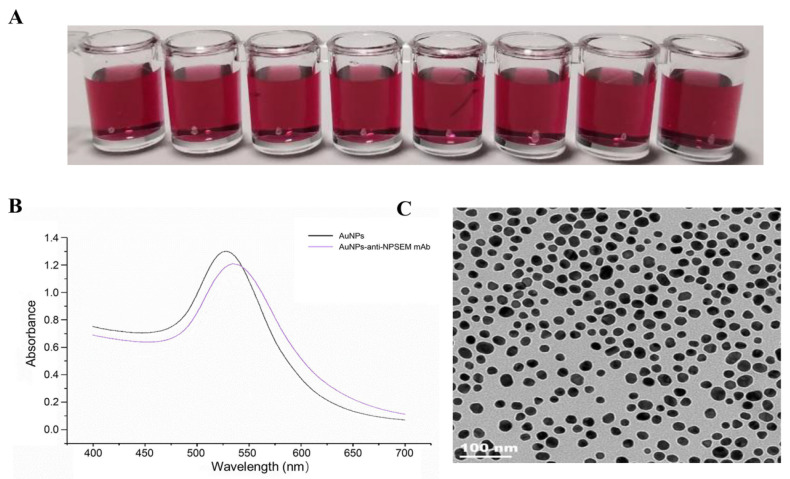
Identification of the prepared AuNPs. (**A**): AuNPs solution in eyes; (**B**): Visible absorption spectrum of naked AuNPs and AuNPs-anti-NPSEM mAb probe, whose concentrations were 4.0 nM and 1.3 mg mL^−1^, respectively; (**C**): Transmission electron micrograph of AuNPs.

The solution of the prepared AuNPs was wine red, clear, and uniform, with good dispersibility and no other insoluble impurities, which preliminarily proved that the preparation of AuNPs was successful (Figure 3A).

AuNPs were characterized by UV-Vis spectroscopy with wavelength ranging from 400 to 700 nm, in which the maximum absorption wavelength was 528 nm (Figure 3B), which is the characteristic absorbance peak of AuNPs, indicating a successful preparation. The average diameter of these uniform particles was about 31.5 nm, according to the linear regression equation: y = 0.4271x + 514.56 [30], in which y is the maximum wavelength of absorption, and x is the diameter of the gold nanoparticles. The peak width of the maximum absorption peak was narrow and symmetrical, indicating that AuNPs were uniform in size and well dispersed.

The transmission electron microscope of AuNPs is shown in Figure 3C, and the particle size of the AuNPs was about 28–33 nm, consistent with the calculation result of the visible light absorption spectrum of the AuNPs. The results of transmission electron microscope and visible light absorption spectrum showed that the preparation of AuNPs was successful.

### 3.3. Optimization and Identification of AuNPs-Labeled Antibody Immunoprobe

In the preparation process of AuNPs-labeled antibody probe, the particle size of colloidal gold, the amount of antibody, and the pH of the labeling system have great effects on the stability and sensitivity of the AuNPs-labeled antibody probe (Table 1). The scanning results of AuNPs-anti-NPSEM mAb by UV-Vis’s spectrophotometer are shown in Figure 3B, whose maximum absorption wavelength had an obvious right shift compared with that of the naked AuNPs. The maximum absorption peak of the AuNPs was 528 nm, and the maximum absorption peaks of the four AuNPs-anti-NPSEM mAb probes was 534.5 nm. The obvious shift of the maximum absorption peak of the AuNPs-anti-NPSEM mAb was due to the increase of the particle size of the antibody adsorbed on the AuNPs surface through electrostatic interaction. At the same time, the maximum absorption peak of the AuNPs-anti-NPSEM mAb probes was narrow and symmetrical, which indicates that the gold labeled antibody probe was stable. This results also verified the successful coupling of the AuNPs-anti-NPSEM mAb probes.

### 3.4. Optimization of the bFQICA

The concentration of AuNPs-anti-NPSEM mAb probes and the amount of the immunoprobes per strip, and the concentration of coat antigen (CPSEM-OVA) and the goat anti-mouse IgG on the NC membrane were investigated (Table 1). The value of IC_50_ was an important parameter for evaluating the bFQICA performance.

### 3.5. Detectability

**Figure 4:**
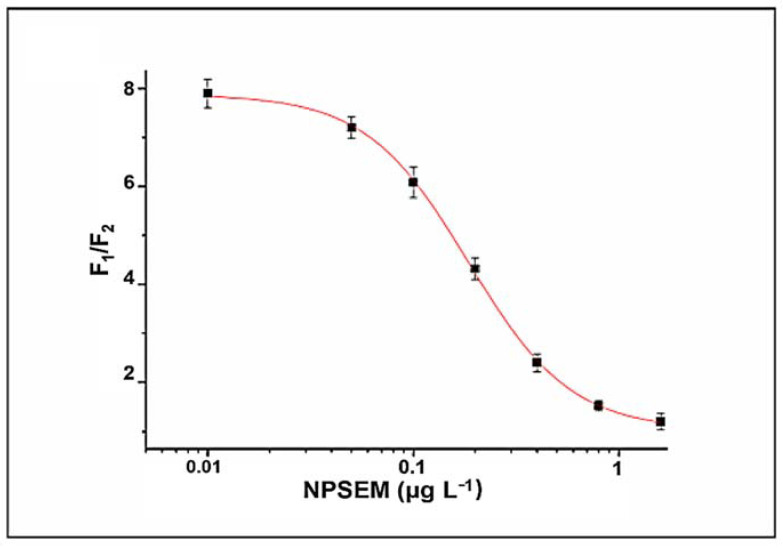
The standard curve of bFQICA method.

SEM was derivatized into NPSEM for detection by the bFQICA. The standard solutions of NPSEM were diluted in PB (0.02 M) to generate the corresponding concentration from 0 to 1.6 μg L^−1^ (0, 0.05, 0.1, 0.2, 0.4, 0.8, 1.6 μg L^−1^). The standard curves were generated with a series of NPSEM solutions. The detectability of the bFQICA was represented by IC_50_ values of 0.19 μg L^−1^ for NPSEM obtained from the standard curves (Figure 4 and Figure S1). The linear range was 0.08–0.41 μg L^−1^, represented by the concentrations causing 20–80% inhibition (Table 1).

In this study, as NFZ was very unstable after entering an animal’s body, it could be quickly metabolized into SEM with smaller molecular weight in a short time and could consequently bind to tissue proteins in a relatively stable state. Because the molecular weight of SEM was too small, a derivatization reagent (2-NBA) was usually used to generate NPSEM, increasing its molecular weight [31]. For animal samples, the matrix component with the greatest interference in the extract was protein. Matrix interferences are a common and challenging problem when applying bFQICA to real samples; therefore, sample pretreatment will directly affect the efficiency and accuracy of detection [32]. The purpose of sample pretreatment is to effectively extract, purify, and concentrate the target analyte and reduce the adverse effect of the matrix effect on immune response as much as possible. Generally, the influence of the matrix effect on immunoassay results can be eliminated or weakened by the dilution method [33,34], which can effectively reduce the proportion of non-specific binding. Separation and extraction are also common methods that can eliminate or reduce the matrix effect by removing or reducing matrix components [35]. In this study, on the basis of common sample pretreatment technology (nitrogen blowing method), the amount of derivatization reagent (0.1 mL, 50 mM 2-NBA) was increased appropriately, the temperature of the derivatization process was increased (60 °C) to achieve rapid derivatization [36], and the sample diluent (0.02 M PBS with 0.05% Tween-20, pH 7.4) was prepared to dilute the extract by 5 times, effectively reducing the matrix effect.

### 3.6. Specificity

There was negligible interference when detecting other chemical substances by the bFQICA (Table S1). The parent nitrofurans, nitrofuran metabolites, and other veterinary drugs commonly used in poultry and aquaculture were individually tested to evaluate the specificity of bFQICA. All the above results indicated the high specificity of the bFQICA for (NP)SEM detection.

### 3.7. Validation of bFQICA

#### 3.7.1. Limit of Detection

The LODs for bFQICA in egg, chicken, fish, and shrimp were 0.09, 0.10, 0.12, and 0.15 μg kg^−1^ for SEM, respectively. The LOD of the developed bFQICA in egg, chicken, fish, and shrimp were below MRPL of 1.0 μg kg^−1^, which is compatible with the EU requirements. The bFQICA method not only had the advantages of being a quantitative method for the detection of SEM compared with the published multi-CGICA method [19], but it also had a wider linear range than that of the published MBs-ICA method in fish samples (0.1–50 μg L^−1^) [37]. Especially, although bFQICA nearly had the detectability of the instrument method using UPLC-MS/MS for SEM [12], it had the advantages of easy operation, low cost, and short implementation time. The developed bFQICA is an improved version of traditional colloidal gold immunochromatography, and it breaks through the bottleneck that AuNPs are usually only suitable for qualitative detection.

#### 3.7.2. Accuracy and Precision

To evaluate the accuracy and precision of the developed bFQICA, blank animal-derived food samples were fortified with SEM at concentrations of LOD, 2LOD, 4LOD, and 1 μg kg^−1^ (MRPL). The recoveries of intra-assay ranged from 75.9% to 104.5%, and the recoveries of inter-assay ranged from 75.7% to 105.1% (Table 2). All the CV values were less than 15%. All the above results confirmed that the bFQICA was an accurate and effective method and that it is fit for the rapid determination of SEM in animal-derived foods.

### 3.8. Application in Field Samples

Eighty field samples of animal-derived food (egg, chicken, fish, and shrimp) were detected by the bFQICA and LC-MS/MS, respectively [14,38]. All the detection results of the two methods were coincident (Table 3), suggesting that the developed bFQICA method was a reliable method for the detection of trace SEM residues in animal-derived foods.

## 4. Conclusions

This is the first report on the bFQICA method for SEM detection. In this study, the bFQICA for the quantitative determination of SEM in animal-derived foods was successfully developed. The LODs for bFQICA in egg, chicken, fish, and shrimp were 0.09, 0.10, 0.12, and 0.15 μg kg^−1^ for SEM, respectively, with the recoveries ranging from 73.5% to 109.2% (CVs < 15%), using a process that only takes 13 min. The analysis of animal-derived food samples by bFQICA was in accordance with that of LC-MS/MS. Compared with the traditional CGICA method, the detectability of the bFQICA method was higher, and the detection time was shortened compared with heterogeneous reactions such as ELISA. In addition, the concentration of SEM can be directly displayed by the built-in QR-code, which is efficient and convenient. As a promising approach, this method could also be extended for the nitrofurans metabolite in aquaculture and poultry products.

## Figures and Tables

**Table 1:** Analytical parameters of the bFQICA for the detection of (NP)SEM.

	Characterization	Results
AuNPs-anti-NPSEM mAb probes (1mL reaction system)	The particle size of AuNPs	30 nm
	pH	8 (0.8% *v*/*v* K_2_CO_3_)
	Anti-NPSEM mAb (μg mL^−1^)	2.55
	Storage buffer	0.02 M PB (0.5% BSA, 0.5% Triton X-100, 5% sucrose, 0.03% NaN_3_, pH 7.4)
Optimum parameters of the established bFQICA (50 μL reaction system)	The dosage of AuNPs-anti-NPSEM mAb probe	4 μL per well
	AuNPs-anti-NPSEM mAb probe (μg mL^−1^)	2.55
	CPSEM-OVA (mg mL^−1^)	0.17
	Concentration of goat anti-mouse IgG (mg mL^−1^)	0.33
	rehydrated solution (μL)	46 (0.02 PB)
Analytical parameters of NPSEM standard curve	IC_50_ (μg L^−1^)	0.19
	20–80% inhibition (μg L^−1^)	0.08–0.41
	LODs (μg kg^−1^)	0.09 (egg), 0.10 (chicken), 0.12 (fish), 0.15 (shrimp)

**Table 2:** Recovery and precision of SEM added in egg, chicken, fish, and shrimp.

Samples	Spiked Concentration (μg kg^−1^)	Intra-Assay ^a^		Inter-Assay ^b^	
		Measure ± SD ^c^ (μg kg^−1^)	Recovery ± CV ^d^ (%)	Measure ± SD (μg kg^−1^)	Recovery ± CV (%)
Egg	0.09	0.074 ± 0.007	82.2 ± 8.9	0.077 ± 0.008	85.8 ± 9.9
	0.18	0.181 ± 0.012	100.6 ± 6.4	0.168 ± 0.017	93.4 ± 10.3
	0.36	0.302 ± 0.011	83.8 ± 3.8	0.323 ± 0.016	89.6 ± 5.1
	1.00	0.981 ± 0.037	98.1 ± 3.8	0.932 ± 0.049	93.2 ± 5.3
Chicken	0.10	0.090 ± 0.006	90.1 ± 7.2	0.078 ± 0.007	78.1 ± 8.5
	0.20	0.167 ± 0.015	83.5 ± 9.1	0.187 ± 0.012	93.6 ± 6.2
	0.40	0.418 ± 0.015	104.5 ± 3.6	0.420 ± 0.016	105.1 ± 3.9
	1.00	1.012 ± 0.050	101.2 ± 4.9	0.946 ± 0.069	94.6 ± 7.3
Fish	0.12	0.091 ± 0.006	75.9 ± 6.9	0.101 ± 0.006	83.9 ± 6.0
	0.24	0.212 ± 0.012	88.4 ± 5.8	0.211 ± 0.018	88.1 ± 8.6
	0.48	0.461 ± 0.034	96.0 ± 7.4	0.478 ± 0.045	99.6 ± 9.4
	1.00	0.858 ± 0.040	85.8 ± 4.7	0.916 ± 0.057	91.6 ± 6.2
Shrimp	0.15	0.118 ± 0.012	78.4 ± 10.2	0.114 ± 0.013	75.7 ± 11.5
	0.30	0.269 ± 0.017	89.8 ± 6.3	0.279 ± 0.025	92.9 ± 8.9
	0.60	0.555 ± 0.049	92.5 ± 8.8	0.609 ± 0.033	101.5 ± 5.4
	1.00	0.880 ± 0.032	88.0 ± 3.6	0.900 ± 0.060	90.0 ± 6.7

^a^ Intra-assay variation was detection by 6 replicates on a single day. ^b^ Inter-assay variation was detection by 6 replicates on 3 different days. ^c^ SD, standard deviation. ^d^ CV, coefficient of variation.

**Table 3:** Determination of SEM in field animal-derived food samples collected by the bFQICA and LC-MS/MS (*n* = 3).

Sample	No.	bFQICA, Mean ± SD (μg kg^−1^)	LC-MS/MS, Mean ± SD (μg kg^−1^)
Egg	1–9	ND ^a^	ND
	10	ND	ND
	11–20	ND	ND
Chicken	1–6	ND	ND
	7	ND	ND
	8–20	ND	ND
Fish	1	0.88 ± 0.04	0.92 ± 0.03
	2–20	ND	ND
Shrimp	1–12	ND	ND
	13	ND	ND
	14–20	ND	ND

^a^ ND not detected.

## Supplementary Materials

The following are available online at https://www.mdpi.com/article/10.3390/foods10071668/s1, Figure S1: Detection of (NPSEM) of gradient concentration by the bFQICA test card based on grey signal of AuNPs by eyes. Table S1: Cross reactivity (CR) of NPSEM and its analogs by bFQICA test cards.
