# Supplementary material for: Quantitative Determination of Nitrofurazone Metabolites in Animal-Derived Foods Based on a Background Fluorescence Quenching Immunochromatographic Assay
Source: Foods. 2021 Jul 20;10(7):1668. doi: 10.3390/foods10071668 (PMC8307092; doi:10.3390/foods10071668)
Supplement: Supplementary file 1 [file foods-10-01668-s001.zip › foods-1270797-supplementary.pdf]

foods

Supplementary Materials

Quantitative Determination of Nitrofurazone Metabolites in Animal-Derived Foods Based on a Background Fluorescence Quenching Immunochromatographic Assay

Yuping Wu [1], Jia Wang [2], Yong Zhou [3], Yonghua Qi [1], Licai Ma [4], Xuannian Wang [1,*] and Xiaoqi Tao [2,*]

1 College of Life Science and Basic Medicine, Xinxiang University, Xinxiang, Henan 453003, China; wuyuping62@xxu.edu.cn (Y.W.); qyh@xxu.edu.cn (Y.Q.)
2 College of Food Science, Southwest University, Chongqing 400715, China; wangjia2020@cau.edu.cn
3 College of Veterinary Medicine, China Agricultural University, Beijing 100193, China; zhouyong@cau.edu.cn
4 Beijing WDWK Biotech Co., Ltd., Beijing 100095, China; malicai@wdwkbio.com
* Correspondence: yuefeng@xxu.edu.cn (X.W.); taoxiaoqi@swu.edu.cn (X.T.); Tel.: +86-15090098008 (X.W.); +86-18306008102 (X.T.)

Supplementary Materials

NFZ, SEM, nitrofurantoin (NFT), furaltadone (FTD), furazolidone (FZD), 1-aminohydantoin (AHD), 3-amino-5-methylmorpholino-2-oxazolidinone (AMOZ), 3-amino-2-oxazolidinone (AOZ), nitrophenyl SME, AHD, AMOZ and AOZ derivatives (NPSEM, NPAHD, NPAMOZ, NPAOZ), Norfloxacin (NOR), Sulfamethazine (SMZ), Penicillin G (PEN), Tetracycline (TC), Chloramphenicol (CAP), Leucomalachite Green (LMG) were obtained from Dr. Ehrenstorfer (Augsburg, Germany). Bovine serum albumin (BSA), 2-nitrobenzaldehyde (2-NBA) and Proclin 300 were obtained from Sigma-Aldrich (St. Louis, MO, USA). Other chemical substances were purchased from Beijing Chemical Reagent Company (Beijing, China). All other chemicals and solvents were analytical reagent grade.

A Milli-Q H_2O system with a resistivity of 18.0 MΩ cm^{-1} and the NC membrane (Nitrocellulose Filter membrane, Millipore 2.0) were supplied by EMD Millipore Corp. (Billerica, MA, USA).

Sample Preparation and Sample Pretreatment (Derivation of SEM)

Briefly, homogenized samples (2.00 ± 0.02 g) were placed into a centrifugal tube (50 mL) followed by adding of 4 mL of deionized water, 0.5 mL of 1 M HCl, and 0.1 mL of 2-NBA (50 mM) in sequence. After vortexed for 3 min, the mixture was incubated in water bath at 60 ℃ for 2 h. Then, 5 mL of K_2HPO_4 (0.1 M), 0.4 mL of NaOH (1 M) and 6 mL ethyl acetate were added to the cooling mixture and centrifuged at 4000 g for 10 min at room temperature after whirling violently for 1 min. Next, 3 mL of supernatant was transferred into a new centrifugal tube (4 mL) and dried with nitrogen gas at 50 ℃. Finally, 2.0 mL N-hexane and 0.02 M PB (pH 7.4) were added to the dried residues, whirling gently for 2 min, and the lower layer solution phase (the nitrophenyl derivatives) was collected by centrifugation at 4000 g (5 min).

In this work, due to the low molecular weight of the NFZ metabolite of SEM, 2-NBA is often used to derivatize the metabolite to increase the molecular weight in the sample pretreatment process before detection. Derivatization technology is to quantitatively convert a target compound in a sample that is difficult to analyze and detect into another compound that is easy to analyze and detect, through which the target compound can be qualitatively and/or quantitatively analyzed. When the analyte of SEM was derivatized with 2-NBA into NPSEM during the sample preparation, the direct result of the bFQICA is the concentration of NP derivative, which can be converted into the concentration of SEM by the following formula (1):

$$C(SEM) = [Mw(SEM)/Mw(NPSEM)] \times C(NPSEM) \quad (1)$$

Standard Curves and Generation of QR-code

For the quantitative assay, standard curves were obtained by plotting F_1/F_2 against the concentration of SEM and fitted to a four-parameter logistic equation using Origin (version 2017, OriginLab, Northampton, MA, USA) software packages, in which F_1/F_2 was defined as the fluorescence intensity ratio of the C line to T line. This is an example of an equation (2):

$$y = (A - D)/[1 + (x / C)^B] + D \quad (2)$$

Where, y is the value of F_1/F_2; x is the concentration of SEM; A is the response value at high asymptote; B is the curve slope at the inflection point; C is the x value at the infection point (corresponding to the half-inhibition concentration (IC_{50}); and D is the response value at low asymptote. Four parameters, A, B, C and D, were input into software (Nice Label Pro 2017) to generate QR-code with the built-in standard curve and the QR-code was printed by barcode printer (Label Shop). The accurate concentration of analytes could be obtained by scanning the QR-code.

Specificity of bFQICA

The parent nitrofurans (NFZ, NFT, FTD, FZD), nitrofuran metabolites (NPAHD, NPAMOZ, NPAOZ), and other veterinary drugs commonly used in livestock and poultry and aquaculture (egg, chicken, fish, and shrimp), such SMZ,

TC, NOR, PEN, CAP, and LMG were individually tested to evaluate the specificity of bFQICA. All of the results indicated the high specificity of the bFQICA for (NP)SEM was acceptable (Table S1).

Validation of bFQICA

For validation of bFQICA, animal-derived food samples were confirmed to be free SEM by LC-MS/MS. The limit of detection (LOD) was calculated by the mean value of 20 blank samples plus three times the mean standard deviation (mean+3SD). The accuracy of the method was represented by recovery, which was calculated as follows:

$$\text{Recovery (\%)} = (\text{Concentration of measured}/\text{Concentration of fortified}) \times 100\% \quad (3)$$

Coefficient of variation (CV) was used to represent the intra-assay and inter-assay precisions of the method. Each sample was evaluated 10 replicates and on 3 consecutive days to verify the repeatability. In addition, each of 20 egg, chicken, fish and shrimp samples were analyzed by the developed bFQICA and LC-MS/MS, respectively.

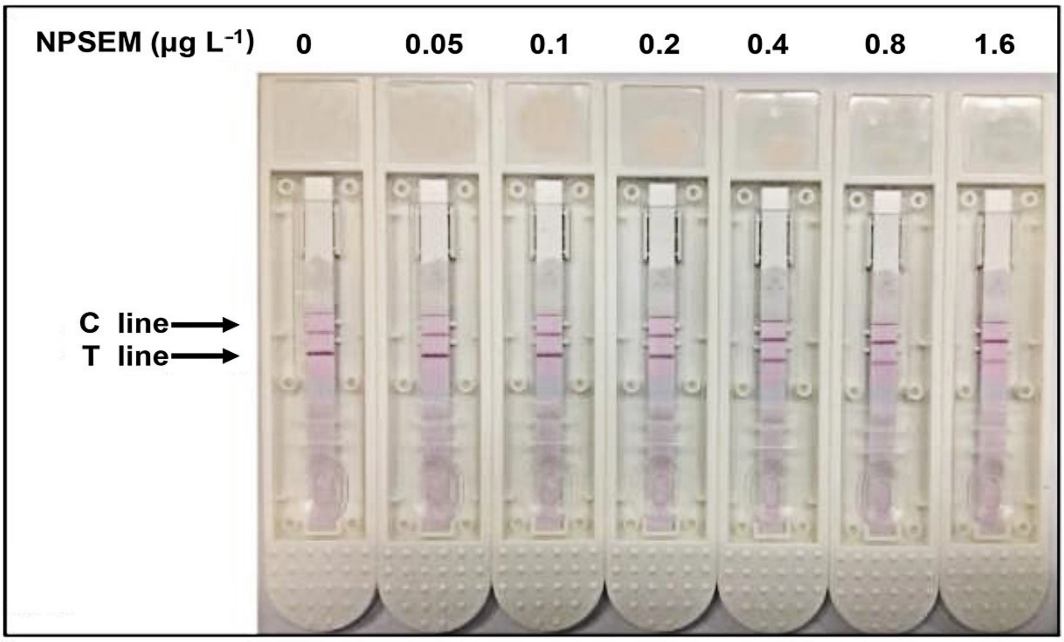

Figure S1. Detection of (NPSEM) of gradient concentration by the bFQICA test card based on grey signal of AuNPs by eyes.

Table S1. Cross reactivity (CR) of NPSEM and its analogs by bFQICA test cards.

NPSEM and its analogues.	IC_{50} (μg L^{-1})	CR (%)
NPSEM	0.19	100%
NPAHD	>1000	<0.02
NPAMOZ	>1000	<0.02
NPAOZ	>1000	<0.02
NFZ	>1000	<0.02
NFT	>1000	<0.02
FTD	>1000	<0.02
FZD	>1000	<0.02
SEM	>1000	<0.02
AHD	>1000	<0.02
AMOZ	>1000	<0.02
AOZ	>1000	<0.02
SMZ	>1000	<0.02
TC	>1000	<0.02
NOR	>1000	<0.02
PEN	>1000	<0.02
CAP	>1000	<0.02
LMG	>1000	<0.02
